# Supplementary material for: Specialist and Generalist Fungal Parasites Induce Distinct Biochemical Changes in the Mandible Muscles of Their Host
Source: Int J Mol Sci. 2019 Sep 17;20(18):4589. doi: 10.3390/ijms20184589 (PMC6769763; doi:10.3390/ijms20184589)
Supplement: Supplementary file 1 [file ijms-20-04589-s001.pdf]

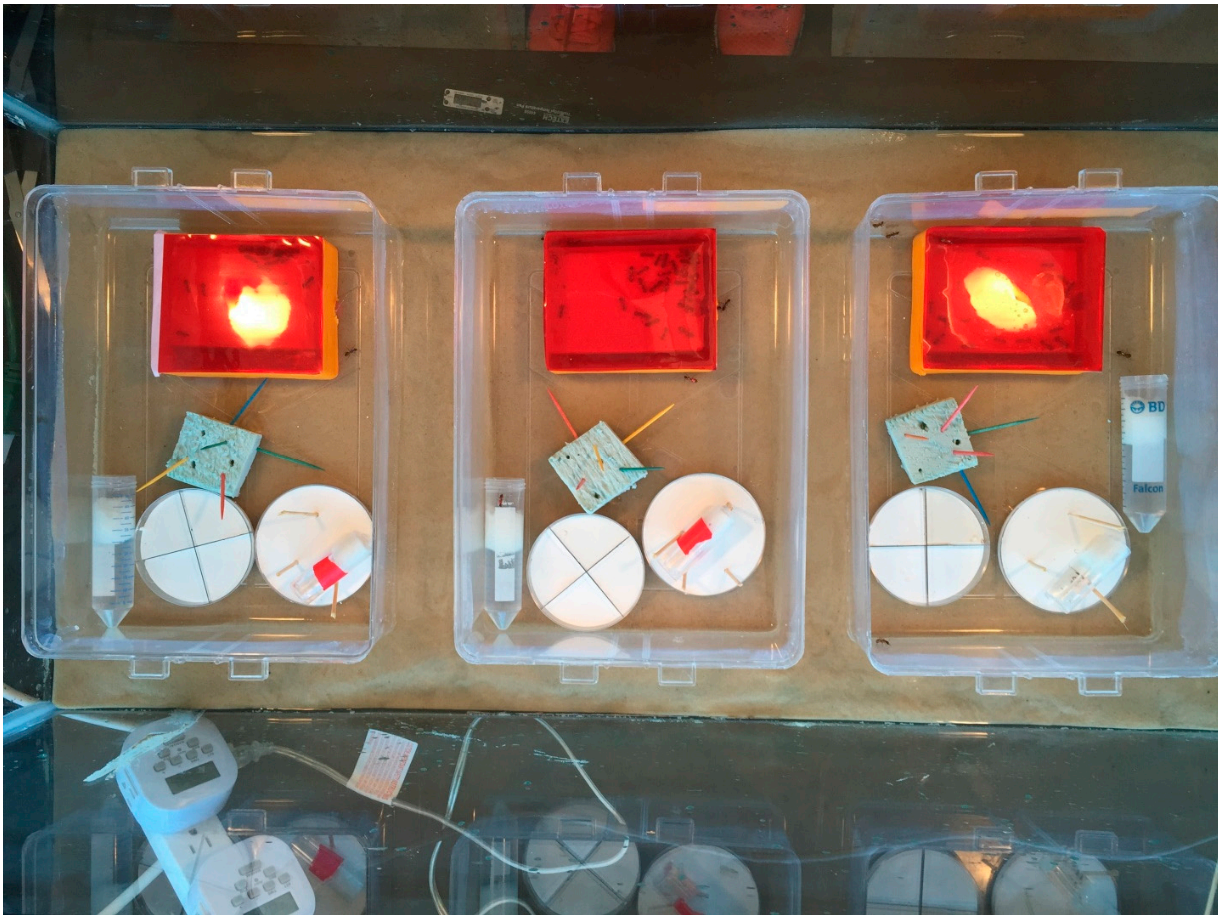

Figure S1: Ants infected with *O. unilateralis*

Table S1: Colony information of samples used for the metabolites study

Colony	KFM1	KFM1	KFM22	KFM22	KFMX	KFM1	KFM1	KFM22	KFMX	KFMX
Sample	MCO1	MCO2	MCO3	MCO4	MCO5	MCO6	MCO7	MCO8	MCO9	MCO10
Colony	KFM1	KFM22	KFMX	KFMX	KFMX	KFM1	KFM1	KFM1	KFMX	KFM22
Sample	MBE1	MBE2	MBE3	MBE4	MBE5	MBE6	MBE7	MBE8	MBE9	MBE10
Colony	KFM1	KFM1	KFMX	KFM1	KFM22	KFM1	KFM1	KFM22	KFM22	KFMX
Sample	MOR1	MOR2	MOR3	MOR4	MOR5	MOR6	MOR7	MOR8	MOR9	MOR10
